# Supplementary material for: Obtaining maximum information from colony counts with REPOP
Source: eLife. 2026 Oct 9;14:RP107122. doi: 10.7554/eLife.107122 (PMC13652539; doi:10.7554/eLife.107122)
Supplement: Supplementary file 1 [file elife-107122-mdarchecklist1.pdf]

Materials Design Analysis Reporting (MDAR) Checklist for Authors

The MDAR framework establishes a minimum set of requirements in transparent reporting mainly applicable to studies in the life sciences.

eLife asks authors to **provide detailed information within their article** to facilitate the interpretation and replication of their work. Authors can also upload supporting materials to comply with relevant reporting guidelines for health-related research (see EQUATOR Network), life science research (see the BioSharing Information Resource), or animal research (see the ARRIVE Guidelines and the STRANGE Framework; for details, see *eLife*'s Journal Policies). Where applicable, authors should refer to any relevant reporting standards materials in this form.

For all that apply, please note **where in the article** the information is provided. Please note that we also collect information about data availability and ethics in the submission form.

Materials:

Newly created materials	Indicate where provided: section/figure legend	N/A
The manuscript includes a dedicated "materials availability statement" providing transparent disclosure about availability of newly created materials including details on how materials can be accessed and describing any restrictions on access.	No newly created research materials requiring distribution are described; software and datasets are covered under Code/Data availability.	X

Antibodies	Indicate where provided: section/figure legend	N/A
For commercial reagents, provide supplier name, catalogue number and RRID, if available.	No antibodies are used.	X

DNA and RNA sequences	Indicate where provided: section/figure legend	N/A
Short novel DNA or RNA including primers, probes: Sequences should be included or deposited in a public repository.	No novel DNA/RNA sequences, primers, or probes are reported.	X

Cell materials	Indicate where provided: section/figure legend	N/A
Cell lines: Provide species information, strain. Provide accession number in repository OR supplier name, catalog number, clone number, OR RRID.	No cell lines or primary cell cultures are used.	X
Primary cultures: Provide species, strain, sex of origin, genetic modification status.		X

Experimental animals	Indicate where provided: section/figure legend	N/A
Laboratory animals or Model organisms: Provide species, strain, sex, age, genetic modification status. Provide accession number in repository OR supplier name, catalog number, clone number, OR RRID.	Appendix 3, Caenorhabditis elegans strain AU37 [glp-4(bn2) I; sek-1(km4) X] was obtained from the Caenorhabditis Genetics Center (CGC).	

Animal observed in or captured from the field: Provide species, sex, and age where possible.	No field animals are used.	X

Plants and microbes	Indicate where provided: section/figure legend	N/A
Plants: provide species and strain, ecotype and cultivar where relevant, unique accession number if available, and source (including location for collected wild specimens).	No plants are used.	X
Microbes: provide species and strain, unique accession number if available, and source.	Appendix 3, Escherichia coli strain OP50 was obtained from the Caenorhabditis Genetics Center (CGC). Culture, media, OD and plating conditions are also reported.	

Human research participants	Indicate where provided: section/figure legend) or state if these demographics were not collected	N/A
If collected and within the bounds of privacy constraints report on age, sex, gender and ethnicity for all study participants.	No human research participants are included.	**X**

Design:

Study protocol	Indicate where provided: section/figure legend	N/A
If the study protocol has been pre-registered, provide DOI. For clinical trials, provide the trial registration number OR cite DOI.	The study is not a clinical trial and no preregistration is reported.	X

Laboratory protocol	Indicate where provided: section/figure legend	N/A
Provide DOI OR other citation details if detailed step-by-step protocols are available.	Appendix 3, C. elegans culture, cites Eisenmann (2005) and Vega & Gore (2017); the latter is specifically cited for worm disruption and intestinal-population plating. E. coli culture/plating steps are described directly in Appendix 3.	

Experimental study design (statistics details) *		
**For in vivo studies: State whether and how the following have been done**	**Indicate where provided: section/figure legend. If it could have been done, but was not, write "not done"**	**N/A**
Sample size determination	Actual experimental sample sizes are stated in Results and Appendix 3 (e.g., 97 total E. coli samples; 25 worms per studied time point). We also show the effects of increasing sample size on synthetic data (Fig3 and Appendix 2)	
Randomisation	Not done	

Blinding	Not done	
Inclusion/exclusion criteria	Methods, discussion of the colony-count cutoff and “Likelihood for Model 3”: plates above the preset countability cutoff are not directly counted, and the likelihood explicitly accounts for this selection. No other inclusion/exclusion criteria are reported.	

**Sample definition and in-laboratory replication**	**Indicate where provided: section/figure legend**	**N/A**
State number of times the experiment was replicated in the laboratory.	Results, “Discerning different true concentrations of bacteria,” and Appendix 3: 97 E. coli samples total; 25 samples from each of the first three OD conditions and 22 from the fourth, with three serial dilutions per sample. Results, C. elegans application: 25 individual worms per time point, each serially diluted on three plates.	
Define whether data describe technical or biological replicates.	C. elegans: individual worms are biological samples; multiple dilution plates are repeated measurements of each worm homogenate. E. coli OD experiment: repeated subsamples are drawn from four source vials and each is plated at three dilutions.	

**Ethics**	**Indicate where provided: section/submission form**	**N/A**
Studies involving human participants: State details of authority granting ethics approval (IRB or equivalent committee(s), provide reference number for approval.	No human participants.	X
Studies involving experimental animals: State details of authority granting ethics approval (IRB or equivalent committee(s), provide reference number for approval.	The only animal model is the invertebrate C. elegans. No ethics-approval statement is included in the manuscript.	X
Studies involving specimen and field samples: State if relevant permits obtained, provide details of authority approving study; if none were required, explain why.	No field-collected specimens or field sampling are reported.	X

**Dual Use Research of Concern (DURC)**	**Indicate where provided: section/submission form**	**N/A**
If study is subject to dual use research of concern regulations, state the authority granting approval and reference number for the regulatory approval.	No DURC-regulated work is indicated.	X

Analysis:

Attrition	Indicate where provided: section/figure legend	N/A
Describe whether exclusion criteria were pre-established. Report if sample or data points were omitted from analysis. If yes, report if this was due to attrition or intentional exclusion and provide justification.	Methods and Introduction describe the preset “uncountable” colony cutoff. Model 3 explicitly incorporates the information from lower-dilution plates that exceed the cutoff instead of treating the selection as ignorable. No other omitted samples/data points are reported.	

Statistics	Indicate where provided: section/figure legend	N/A
Describe statistical tests used and justify choice of tests.	Methods: Bayesian inference; population-versus-measurement stochasticity; Gaussian and non-parametric Gaussian-mixture population models; likelihoods for Models 1-3; MAP optimization. Appendix 1 gives prior choices. Appendix 2 reports relative error and Kullback-Leibler divergence. No null-hypothesis significance tests are used.	

Data availability	Indicate where provided: section/submission form	N/A
For newly created and reused datasets, the manuscript includes a data availability statement that provides details for access (or notes restrictions on access).	Data availability section: “The plate counting real data” is linked from the online article.	
When newly created datasets are publicly available, provide accession number in repository OR DOI and licensing details where available.	Data availability section provides a link to the GitHub repository containing the data.	
If reused data is publicly available provide accession number in repository OR DOI, OR URL, OR citation.	The analyses use newly generated data; no reused external dataset is identified	X

Code availability	Indicate where provided: section/figure legend	N/A
For any computer code/software/mathematical algorithms essential for replicating the main findings of the study, whether newly generated or re-used, the manuscript includes a data availability statement that provides details for access or notes restrictions.	Abstract and Introduction: REPOP is publicly available at github.com/LabPresse/REPOP; the repository contains the package, tutorial, scripts and data folders used for manuscript figures	
Where newly generated code is publicly available, provide accession number in repository, OR DOI OR URL and licensing details where available. State any restrictions on code availability or accessibility.	Introduction and reference “Pessoa, 2025”; public GitHub repository LabPresse/REPOP. The repository contains a LICENSE file.	

If reused code is publicly available provide accession number in repository OR DOI OR URL, OR citation.	PyTorch and other dependencies are cited/described, but this checklist item concerns reused code essential to reproduce the main findings; no separate reused analysis code repository is identified	X

Reporting:

The MDAR framework recommends adoption of discipline-specific guidelines, established and endorsed through community initiatives.

**Adherence to community standards**	**Indicate where provided: section/figure legend**	**N/A**
State if relevant guidelines (e.g., ICMJE, MIBBI, ARRIVE, STRANGE) have been followed, and whether a checklist (e.g., CONSORT, PRISMA, ARRIVE) is provided with the manuscript.	No discipline-specific reporting guideline (e.g., ARRIVE, CONSORT, PRISMA) is identified as applicable in the manuscript.	X
