# Supplementary material for: Obtaining maximum information from colony counts with REPOP
Source: eLife. 2026 Oct 9;14:RP107122. doi: 10.7554/eLife.107122 (PMC13652539; doi:10.7554/eLife.107122)
Supplement: Peer review history

# Editor report: eLife Assessment

**DOI:** 10.7554/eLife.107122.3.sa0

**Authors:** Anne-Florence Bitbol

**Affiliations:** Ecole Polytechnique Federale de Lausanne (EPFL) Switzerland

**Keywords:** Compelling, Important

This **important** study introduces a Bayesian method to determine bacterial counts that accounts for the experimental noise inherent to dilution and plating methods and distinguishes it from biological uncertainty. The evidence supporting the conclusions is **compelling**, combining simulated data and experimental data. The method will be of interest to microbial ecologists, and potentially to the broader community interested in inference from biological data, even more so if the domain of application and the limitations are further clarified.

# Referee report: Reviewer #1 (Public review)

**DOI:** 10.7554/eLife.107122.3.sa1

Summary:

The authors developed a novel theoretical/computational procedure to count bacterial populations without introducing artificial randomness effects due to dilution. Surprisingly, this very important aspect of studies of bacterial systems has been overlooked. The proposed method provides a simple and transparent approach to eliminate the randomness of bacterial accounting procedures, allowing now to fully concentrate on the intrinsic effects of the studied systems.

Strengths:

A very simple and clear procedure is introduced and explained in full detail. This elegant approach finds an excellent compromise between mathematical rigor and computational efficiency, which is important for practical applications. The provided examples are convincing beyond a doubt, clearly indicating the potential strong impact of the proposed framework. Various complications and possible issues are also discussed and analyzed. This seems to be a very powerful novel method that should significantly advance the analysis of complex biological systems.

Weaknesses:

The only minor weakness that I found is the assumption of independence of bacterial species, which is expressed as the well-stirred approximation. One could imagine that bacterial species might cooperate, leading to non-uniform distributions that are readerivedl. How to distinguish such situations?

I believe that this method can be extended to determine if this is the case or not before the application. For example, if the bacteria species are independent of each other and one can use the binomial distributions - then the Fano factor would be proportional to the overall relative fraction of bacterial species. Maybe a simple test can be added to test it before the application of REPOP. However, I believe that this is a minor issue.

Comments on revised version.

I am satisfied with the correction proposed by the authors. The method is already quite impressive, and there is no need to complicate it at this stage.

# Referee report: Reviewer #2 (Public review)

**DOI:** 10.7554/eLife.107122.3.sa2

I appreciate the thorough responses from the authors, which address my concerns. The expansion of Appendix B as well as the addition of text discussing how the REPOP method interacts with data collection efforts are very useful. These new sections show that relative error decreases with increasing samples, as expected, yet these error metrics, including KL divergence, describing the fit of the full distributions not just the modes, drop off fairly quickly with increasing number of samples showing that REPOP likely minimizes discrepancies between estimated and true distributions even at lower sampling efforts.

Additionally, the extension of the REPOP method to the quantification of multiple bacterial species or phenotypes shows the potential utility of the method in contexts beyond basic plate counts. Between this example and the additional information on how to implement REPOP, I believe this workflow will be attractive and accessible to the audience.

# Author comment: Author response

**DOI:** 10.7554/eLife.107122.3.sa3

**Authors:** Pedro Pessoa, Carol Yunxiao Slayton, Stanimir Asenov Tashev, Rory Kruithoff, Doug P Shepherd, Steve Presse

**Affiliations:** Arizona State University Tempe United States; Arizona State University Tempe United States; Arizona State University Tempe United States; Arizona State University Tempe United States; Arizona State University Tempe United States; Arizona State University Tempe United States

The following is the authors’ response to the original reviews.

**Public Reviews:**

**Reviewer #1 (Public review):**

(R1C1) The only minor weakness that I found is the assumption of independence of bacterial species, which is expressed as the well-stirred approximation. One could imagine that bacterial species might cooperate, leading to non-uniform distributions that are real. How to distinguish such situations?

I believe that this method can be extended to determine if this is the case or not before the application. For example, if the bacteria species are independent of each other and one can use the binomial distributions, then the Fano factor would be proportional to the overall relative fraction of bacterial species. Maybe a simple test can be added to test it before the application of REPOP. However, I believe that this is a minor issue.

This is an interesting point raised by the reviewer.

First, we need to clarify an important point: we do not make a well-stirred assumption. Samples can be drawn and plated from any region of space however small and that region’s population can be quantified using our method. The stirring only occurs after we collect a sample in order to dilute the contents and pour the solution homogeneously over the plate.

As such, learning multiple independent species is possible and not impacted by the dilution (“well-stirred” assumption). In the new first paragraph of the methods section, we made it clear that this assumption concerns the dilution process. REPOP is designed to recover the true underlying heterogeneity in species abundance (even from limited data) by leveraging a Bayesian framework that remains valid regardless of whether species are independent or correlated.

If the method is applied to multiple species as currently implemented, REPOP can recover the marginal distribution of each species, provided that the species are either selectively cultured or produce sufficiently distinguishable colonies on the same plate. To demonstrate this, we have added a new Results subsection with a synthetic two-species example in which the species abundances are correlated across samples.

However, in order to learn the joint distribution and capture correlations between species within samples, the method would need to be extended. At present, in Eq. 5 we sum the likelihood over all values of *n*, using a data-driven cutoff (twice the largest naïvely estimated count times the dilution factor). Extending this to multiple species adding up to (*n*_1_,*n*_2_), while retain the generality of the method, would require quadratically scaling memory with this cutoff in the population number. For this reason while we comment on this in the new paragraph in the conclusion, it is not implemented as part of REPOP.

**Reviewer #2 (Public review):**

(R2C1) A more thorough discussion of when and by how much estimated microbial population abundance distributions differ from the ground truth would be helpful in determining the best practices for applying this method. Not only would this allow researchers to understand the sampling effort necessary to achieve the results presented here, but it would also contextualize the experimental results presented in the paper. Particularly, there is a disconnect between the discussion of the large sample sizes necessary to achieve accurate multimodal distribution estimates and the small sample sizes used in both experiments.

That is a great suggestion from the reviewer. To address it, we expanded Appendix B. We know report (1) the relative error in the estimated means (as already done for Fig. 4 formally 3), and (2) the Kullback-Leibler (KL) divergence between the reconstructed and ground-truth distributions. These metrics will are show as a function of the size of the dataset, for the examples in Fig 3. enabling a direct assessment of how the sampling effort affects the precision of the inference.

That said, we now highlight in the Conclusion that, by explicitly modeling the dilution process within a Bayesian framework, REPOP extracts the maximum information available from each individual sample at a given sample size. This strategy therefore enables more accurate inference with fewer measurements, which is particularly important in applications such as plate counting, where data acquisition is labour-intensive.

**Reviewer #3 (Public review):**

(R3C1) While the study is promising, there are a few areas where the paper could be strengthened to increase its impact and usability. First, the extent to which dilution and plating introduce noise is not fully explored. Could this noise significantly affect experimental conclusions? And under what conditions does it matter most? Does it depend on experimental design or specific parameter values? Clarifying this would help readers appreciate when and why REPOP should be used.

We agree with the reviewer that this is an important point, and we expanded Appendix B to include a quantitative analysis using simulated data (Fig. 3, formely 2), reporting both relative error and KL divergence as a function of dataset size. This complements our response to R2C1 clarifying when REPOP offers the greatest benefit.

In addition, we will expand the discussion on how modeling dilution noise becomes essential when learning population dynamics. In particular, we emphasize? the role of Model 3, especially relevant when working with multiple plates and approaching the asymptotic regime; an aspect that was alluded to in Fig. 3 but not fully explored.

(R3C2) Second, more practical details about the tool itself would be very helpful. Simply stating that it is available on GitHub may not be enough. Readers will want to know what programming language it uses, what the input data should look like, and ideally, see a step-by-step diagram of the workflow. Packaging the tool as an easy-to-use resource, perhaps even submitting it to CRAN or including example scripts, would go a long way, especially since microbiologists tend to favor user-friendly, recipe-like solutions.

In the new paragraphs of the introduction, we made clear that REPOP is written in Python (PyTorch), installable via pip, and designed for ease of use. We are also expanding the tutorials to include clearer guidance on data formatting and common workflows. The new workflow figure (Fig 2) better illustrates the full process.

(R3C3) Third, it would be great to see the method tested on existing datasets, such as those from Nic Vega and Jeff Gore (2017), which explore how colonization frequency impacts abundance fluctuation distributions. Even if the general conclusions remain unchanged, showing that REPOP can better match observed patterns would strengthen the paper’s real-world relevance.

We thank the reviewer for this interesting suggestion. We agree that applying REPOP to additional existing datasets would make REPOP’s relevance clearer. However, the Vega and Gore datasets lack the information required. REPOP requires the plate count measurement process to be specified, including the dilution factors used for each measurement. Furthermore, we can leverage on additional information about the experimental procedure when the colony cutoffs and dilution schedules used are reported. Without the dilution factors, the likelihood connecting the observed colony counts to the underlying population size is not possible. We hope this clarification will help make future datasets made available publicly more useful for purposes of uncertainty propagation.

(R3C4) Lastly, it would be helpful for the authors to briefly discuss the limitations of their method, as no approach is without its constraints. Acknowledging these would provide a more balanced and transparent perspective.

We agree with the reviewer. We have added two new paragraphs to the conclusion highlighting important current constraints and future development directions of the framework. In particular, we now discuss that, in its present implementation, REPOP focuses on the population distribution that maximizes the posterior, rather than returning posterior uncertainty over the reconstructed distributions themselves. We also note the computational demands of the method, making GPU acceleration highly beneficial and more complex multi-population inference computationally challenging. This discussion synthesizes points raised throughout our response to R1C1 and the reviewers and provides a more balanced perspective on the current scope of the method.
